# Digital Parenting of Emerging Adults in the 21st Century

**DOI:** 10.3390/socsci10120482

**Published:** 2021-12-16

**Authors:** Michaeline Jensen, Andrea M. Hussong, Emily Haston

**Affiliations:** 1 Department of Psychology, University of North Carolina at Greensboro, Greensboro, NC 27402, USA; 2 Department of Psychology and Neuroscience, University of North Carolina at Chapel Hill, Chapel Hill, NC 27599, USA

**Keywords:** text messaging, parent-child relationship, communication, mobile phones, interactions, parenting, emerging adulthood

## Abstract

In emerging adulthood, when many young people are away from their families for the first time, mobile phones become an important conduit for maintaining relationships with parents. Yet, objective assessment of the content and frequency of text messaging between emerging adults and their parents is lacking in much of the research to date. We collected two weeks of text messages exchanged between U.S. college students (*N* = 238) and their parents, which yielded nearly 30,000 parent-emerging adult text messages. We coded these text message exchanges for traditional features of parent-emerging adult communication indexing positive connection, monitoring and disclosures. Emerging adults texted more with mothers than with fathers and many messages constitute parental check-ins and emerging adult sharing regarding youth behavior and well-being. Findings highlight that both the frequency and content of parent-emerging adult text messages can be linked with positive (perceived text message support) and negative (perceived digital pressure) aspects of the parent-emerging adult relationship. The content of parent-emerging adult text messages offers a valuable, objective window into the nature of the parent-emerging adult relationships in the digital age of the 21st century.

## 1. Introduction

According to classic theories from the last century, adolescence lays at the nexus of increasing needs for both independence and ongoing support from parents, a developmental tension thought to resolve with adulthood (Erikson 1968; Franz and White 1985; Ryan and Lynch 1989). Twenty-first century scholars, however, now recognize that fulfilling and balancing these developmental needs extends into emerging adulthood, a developmental period between adolescence and adulthood (Arnett 2014). Indeed, the majority of emerging adults today do not describe themselves as fully mature adults who have achieved such developmental milestones and their parents tend to agree (Arnett 2000; Arnett and Schwab 2013). As a result, emerging adulthood marks the start of a second lap for the parent-child relationship, one in which both parents and emerging adults must accommodate shifting roles, priorities, and interaction styles (Mullendore et al. 2018), often through the use of modalities uncommon in the prior century.

Most notably, in the digital age of the twenty-first century, parent-emerging adult interactions are increasingly virtual (Jennings and Wartella 2012). Families have more ways to stay in touch over the course of the day than ever before. Most parents can send their emerging adult a text or call at a moment’s notice, and emerging adults likewise have a line to parental support and guidance constantly at their fingertips. Yet, we still have much to learn about how and how much families interact digitally and the extent to which the frequency and content of digital interactions may help or hinder the delicate balance between autonomy and parent-child relatedness in emerging adulthood.

Therefore, the present study extends existing literature which points to the mobile phone as a new tool for digitally enacting traditional parenting and parent-child interaction behaviors (Jensen et al. 2021; Fletcher et al. 2018; Rudi and Dworkin 2018). We examine how and how much parent-emerging adult dyads digitally engage in two key forms of parent- child behaviors that reflect (a) positive connection and (b) monitoring and disclosing behaviors. Further, we test whether established associations between these offline parenting behaviors and autonomy and relatedness are evident in the digital world of emerging adults.

### 1.1. Parenting in Emerging Adulthood

Despite half a century of research on what parenting behaviors most facilitate social and emotional development in children and adolescents (Baumrind 1966; Maccoby and Martin 1983; Schaefer 1965), research on the parenting of emerging adults has only recently begun (Padilla-Walker and Nelson 2019). Although lacking a unifying theory, research on the parenting of emerging adults often encompasses several behaviors consistently linked with well-being across development (Hussong et al. n.d.; Padilla-Walker and Nelson 2019). These include behaviors aligned with classic parenting styles (Baumrind 1966, 1991) such as parental engagement in *positive connection* (which encompasses constructs like warmth, support, sensitivity, and responsiveness) and *parental monitoring* of child behavior and related child disclosures (which includes limit setting, structure, demandingness and control; Schaefer 1965; McKee et al. 2009; Collins et al. 2000).

#### 1.1.1. Positive Connection in Parent-Emerging Adult Relationships

The fundamental importance of parental positive connection to adaptive development and indeed survival is well established in childhood through studies on parental sensitivity and attachment (Cox and Harter 2003). Although we know that time spent together tends to decline from preadolescence through late adolescence (Larson et al. 1996), perceived parental support seems to decline from early to mid-adolescence but then rise again from mid to late adolescence (De Goede et al. 2009). Overall, research suggests that parents continue to be an important source of positive support throughout adolescence and even into emerging adulthood (O’Connor et al. 1996; Swartz et al. 2011), with stronger indicators of positive connections with mothers than fathers (Nelson et al. 2011). Further, parental support of emerging adults has become increasingly prevalent over historical time (Eggebeen 1992). Parental support may be especially important in successful transitions out of the family home (Hussong and Chassin 2004), including among emerging adults who leave to attend college (Fingerman et al. 2012). Many scholars include parental provision of social support as a dimension of positive parent-child connection (Furman and Buhrmester 1985), which can include forms of nurturant (emotional and/or esteem), informational (or advice), and tangible (or instrumental) support (Barrera and Ainlay 1983; Cutrona and Russell 1990). Recent research highlights the importance of parsing the emerging adult’s level of desired support (and for what type) relative to the quantity and type of support provided by their parent (Wang 2019). Across these literatures, a positive, connected, supportive parent-emerging adult relationship is clearly a valuable resource that can bolster healthy emerging adult relatedness, well-being and adjustment (Barry et al. 2008; Fingerman et al. 2012; Padilla-Walker and Nelson 2019).

#### 1.1.2. Parental Monitoring and Youth Disclosures in Parent-Emerging Adult Relationships

Historically aligned with parental structure and limit setting, parental monitoring is viewed as a protective factor (especially against externalizing problems) in adolescence (Dishion et al. 1991; Galambos et al. 2003; Gray and Steinberg 1999; Kurdek and Fine 1994). Consistent with a stage-environment fit perspective, parental monitoring and its protective benefits may wane as adolescents become emerging adults, given growing independence and self-reliance (Eccles et al. 1993). Indeed, high levels of parental monitoring in emerging adulthood ought to undermine autonomy development (Padilla-Walker and Nelson 2019). Interestingly, however, most emerging adults today believe that their parents have legitimate authority to monitor and prescribe behavior in at least some aspects of their lives (Padilla-Walker et al. 2014). Current conceptualizations of monitoring distinguish between different forms of this behavior (i.e., parental control, rules, and solicitation of information) as well as recognize the role of youth in disclosing information as part of how parents’ gain the knowledge used to monitor children (Stattin and Kerr 2000). Research in adolescence suggests that mothers are involved in more solicitation and control as well as receive more disclosures about youth activities as compared to fathers (Smetana et al. 2006; Keijsers et al. 2009). Although few studies have parsed the frequency or impact of these specific dimensions of monitoring in emerging adulthood, recent research suggests that emerging adults also disclose more to mothers than fathers, with frequent disclosers (to either parent) enjoying more parental support for their autonomy (Son and Padilla-Walker 2021).

#### 1.1.3. Parenting in Emerging Adulthood as a Two-Way Street

Within studies on the parenting of emerging adults, the *active role of the emerging adult* in shaping interactions and the evolving parent-emerging adult relationship has been largely overlooked (Padilla-Walker and Nelson 2019). Although some research points to the potential for the behavior of older adolescents to evoke parenting behaviors (e.g., Maggs and Galambos 1993; Padilla-Walker et al. 2012), more research is needed to better understand the dynamic interplay between parents and emerging adults. As noted, research on parental monitoring increasingly highlights the importance of distinguishing between parental solicitations and youth disclosures about their own behavior, with disclosures serving as stronger predictors of parental knowledge and youth behavior (Hamza and Willoughby 2011; Urry et al. 2011). Further, scholars have made the distinction between perceived and received (or enacted) social supports (Wills and Shinar 2000), recognizing the role of emerging adults in the seeking and receiving of supports as well. Although “parenting” has most often been conceptualized as things the *parent does or says*, it is imperative to also consider what the *emerging adult child does or says* in order to fully understand how positive connection and monitoring and disclosing behaviors manifest in relationships between emerging adults and their parents.

### 1.2. Parenting in the Digital Age

The new millennium has seen frequent contact between college students and their parents, with 40% of students reporting daily interactions with family members and 82% reporting contact at least weekly (Liu et al. 2008). College students report that they call or text with their mothers about 12 times per week and their fathers 6 times per week (Miller-Ott et al. 2014). A total of 35% of US parents report that technology makes parenting easier (Lauricella et al. 2016) and many youths likewise tout the benefits of parent-child digital communication (Campbell 2006; Chen and Katz 2009). In addition, more frequent parent-youth digital communication is associated with greater parent-child closeness (Manago et al. 2020), improved health outcomes and less binge drinking (Small et al. 2011, 2013), and better youth self-esteem (Weisskirch 2011). Thus, parenting seems to be facilitated by digital communication (Walker and Rudi 2014). Yet, we know little about how traditional parenting behaviors are enacted within digital contexts.

#### 1.2.1. Parent-Emerging Adult Positive Connection and Monitoring and Disclosing Behaviors via Mobile Phone

Co-construction theory (Subrahmanyam et al. 2006) asserts that youth co-create their online and offline interactions and environments to best suit their developmental needs. Given this intricate intertwining of the online and offline spheres, it is likely that salient aspects of traditional face-to-face parent-emerging adult interactions will manifest digitally. Indeed, the traditional tasks of parenting are evident in parent-adolescent digital communication. Youth and their parents self-report using mobile phones to facilitate both positive connection (i.e., to seek help, receive support, and share experiences with their parents; Chen and Katz 2009) and to engage in monitoring and disclosing behaviors (i.e., for parents to check in and inquire about the youth’s activities and whereabouts; Fletcher et al. 2018; Kasesniemi and Rautiainen 2002; Racz et al. 2017). Using an ecological momentary assessment design, our own research suggests that phone contacts between younger adolescents and parents, though somewhat infrequent, include responding to adolescent mental health needs with both monitoring and support (Jensen et al. 2021). For emerging adults, qualitative interviews (Platt et al. 2014), focus groups (Chen and Katz 2009), and quantitative self-report surveys (Ramsey et al. 2013; Miller-Ott et al. 2014) highlight that digital communication plays a key role in maintenance and evolution of a positive, connected parent-child relationship in the college years. Yet, the way in which positive connection as well as monitoring and disclosure behaviors are enacted as dyadic processes within digital communication between parents and their emerging adults remains to be charted as does the importance of these virtual interactions in supporting autonomy and relatedness in emerging adults.

#### 1.2.2. Text Messaging and Digital Analogues to Autonomy and Relatedness in Emerging Adulthood

One way to gauge the importance of digital interactions between emerging adults and their parents is to evaluate the role such interactions play in fulfilling key functions of traditional parenting behaviors that support development gains. Notably, we would have greater confidence in the primacy of digital forms of building positive connection as well as monitoring and disclosing behaviors if they are associated with indicators of autonomy and relatedness with parents (Ryan and Deci 2000), particularly as manifested in a digital context.

Relatedness is often conceptualized as encompassing constructs such as belonging and attachment and is often operationalized as relationship quality, attachment security, and quality of interactions (Baumeister and Leary 1995; River et al. 2021). Importantly, relatedness in these measures reflects a child’s perception that others, or in this case parents, care about them, support them, and are present in their lives. Digital analogues of relatedness then should reflect these same perceptions in emerging adults. For example, emerging adults may evidence a greater sense of relatedness with parents by viewing digital communications with parents as more supportive. Similarly, more frequent text communication with parents may signal that emerging adults view their parents as more available and present in their lives. Consistent with this view, more frequent calls and texts are linked with student perceptions of more uplifting, supportive, intimate, and satisfying parent-child relationships (Gentzler et al. 2011; Jensen et al. 2021). There is also some evidence that days marked by digital social support and (some forms of) monitoring are described by younger adolescents as involving more positive offline interactions with parents (Jensen et al. 2021). Thus, youth perceptions of parents as supportive via text messaging might translate into a broader sense of relatedness in the parent-emerging adult relationship.

Autonomy involves establishing self-sufficiency and independence. Parents can support autonomy in emerging adults by promoting independence through physical and social distancing as well as by promoting volitional functioning (Benito-Gomez et al. 2020). On the other hand, parents may inhibit autonomy by being too controlling and intrusive, with limited distancing. Parental intrusiveness as an impediment to autonomy in emerging adults has a direct analogue in the digital world. Indeed, the “always on” nature of the mobile phone may facilitate excessive contact that is perceived as autonomy inhibiting, invasive, and privacy-violating (Jensen-Racz et al. 2017). Early studies concur, suggesting that parent phone contacts may be perceived as intrusive (Green 2007; Ling and Yttri 2002), especially when phone contacts are parent- rather than child-initiated (Weisskirch 2009, 2011), and infringe on personal time and space (Williams and Williams 2005). Thus, emerging adults who experience pressure to be digitally available to parents may also view parents as inhibiting their autonomy.

Digital communication between parents and emerging adults may carry both benefits and risks for the evolution of relatedness and autonomy within this relationship and developmental period. Just as there are digital ways of expressing positive connection and monitoring and disclosing behaviors within parent-emerging adult relationships, digital analogues may also be found for how emerging adults experience relatedness and autonomy within their relationships with parents.

#### 1.2.3. Beyond Self-Reports of Digital Communication

Scholars have called for more research that delves into the treasure trove of naturalistic interactions archived within our smartphones (e.g., Reeves et al. 2020). Although recent studies have collected objective data about text message frequency and (to some extent) content in small samples of college students and other young adults, both naturalistically (Aledavood et al. 2016; Eshghinejad and Moini 2016; Ouellette and Michaud 2016) and in the lab (Holtzman et al. 2017), none have yet examined parent-emerging adult text messages nor how these interactions relate to the larger parent-emerging adult relationship. Indeed, the only published study to date which explicitly examines the objective content of parent-child text communications comes from the well-designed Blackberry Project (Underwood et al. 2012). These researchers collected all text messages exchanged (with all relationship partners, including parents) by about 200 adolescents over their entire high school careers (2008–2012). The researchers used a qualitative hand-coding approach to capture antisocial content (e.g., discussions about drugs, aggression, or rule-breaking), negative talk (e.g., negative social interactions, social exclusion, negative appraisals of self or others, expression of negative affect, and sarcasm), positive talk (e.g., discussion of positive events or feelings, positive assessment of self or others), and sexual content (e.g., references to past, present, or future sexual behavior) in text messages taken from 4 days per year (across 4 years) for each adolescent (interrater reliabilities (kappa) ranged from 0.65 to 0.82 across codes; Ehrenreich et al. 2020). Results indicate that teens text far less with parents than with peers; the average participant exchanged 27.58 (*SD* = 27.73) text messages with parents across 4 days in the 12th grade (which comprise about 6.45% of all texts exchanged; *SD* = 8.65). Unsurprisingly, parent-child texts were much more likely to include positive and neutral content than negative, antisocial, and sexual content. This coding scheme was applied to all types of adolescent interactions with all interaction partners, and thus some codes were less relevant to the parent-child relationship. Content analysis that focuses specifically on theoretically driven, parent-child specific, communication processes could help shed light on important dynamics in parenting in the digital age.

### 1.3. The Present Study

The present study directly examined the content of text messages exchanged by parents and emerging adults. We used a qualitative coding scheme to capture theoretically salient aspects of parenting: positive connection and monitoring and disclosure. We also examined associations between these aspects of texting-based interactions and emerging adults’ perceived pressure to engage digitally with parents (a digital analogue to reduced autonomy-granting) and perceived support by parents (a digital analogue to relatedness to parents). To do so, we analyzed data from 238 U.S. college students who permitted sent and received text message downloads from their personal phones from the prior two weeks in 2014–2015. We focus here on objective assessment of the content of both sent and received messages exchanged within parent-emerging adult dyads, allowing us to study the dyadic nature of “positive connection” and “monitoring and disclosing behaviors” in such exchanges.

We used these rich, naturalistic observations of digital interactions between parents and emerging adults to address four aims. First, we sought to describe the overall *frequency* of parent-emerging adult text message interactions. Given the history of digital divides in access to and use of modern communication technologies due to social class (Norris 2001; George et al. 2020), age and gender (Jensen et al. 2021; Rudi et al. 2015), we explored whether these patterns differed based on emerging adults’ gender, race/ethnicity, age, and socioeconomic status. (*Q1: How often are parent-emerging adult dyads engaging in text messaging and are there systematic differences in parent-emerging adult texting frequency?)*. Second, we sought to describe the *content* of parent-emerging adult text interactions, focusing on exchanges reflecting positive connection (warmth, gratitude, and support provided and solicited) and monitoring (parental control and solicitations alongside emerging adult disclosures). (*Q2: In what ways (and to what extent) are parent-emerging adult dyads using text messaging for positive connection and monitoring and disclosing behaviors, and are there differences in the frequency of text messaging for these purposes between mother-emerging adult and father-emerging adult dyads*)

Next, we tested whether the quantity and content of parent-emerging adult text interactions were associated with digitally analogues to autonomy and relatedness. (*Q3: Are parent-emerging adult text frequency and content related to perceived digital pressure and support from parents?*). We hypothesized that emerging adults would perceive greater digital pressure from parents if they exchanged more frequent text messaging and received more texts reflecting parental monitoring (solicitations and control, though not disclosures). We also hypothesized that emerging adults would perceive more support in parents text messages if they exchanged more frequent text messages and exchanged more texts reflecting positive connection. Given higher rates of mother than father communication with emerging adults (Fingerman et al. 2012), we analyzed mother- and father-emerging adult dyads separately, and hypothesized that texting frequency, positive connection, and monitoring and disclosing behaviors would all be more common in mother- than father-emerging adult dyads. Based on prior research, we did not make specific predictions about differential associations of text message frequency and content with digital pressure and text message support between mother- and father-emerging adult dyads (Padilla-Walker and Nelson 2019).

## 2. Materials and Methods

### 2.1. Sample and Procedures

An in-depth description of study procedures can be found in (Hussong et al. 2021). Briefly, participants were drawn from the Real-U Study of College Life (approved by IRB #14–0360) and were originally recruited through email invitations sent to randomly sampled undergraduates at a southeastern U.S. university in 2014–2015 (with oversampling for males and African American students; n = 9000) and through word-of-mouth (n = 57; Hussong et al. 2021). Of these, 1141 were pre-screened as eligible (reporting past year alcohol use to meet the aims of the parent study) with 854 students completing the first visit and 840 completing both visits before study closure. This sample (which comprises college students who report at least some past year drinking) differs somewhat from the population from which it was drawn (where 75% of students report past-year alcohol use; High-Risk Alcohol and Substance Abuse Working Group 2015), though concerns about generalizability are allayed somewhat by the extent to which this sample of 854 participants was highly representative of the student body from which it is drawn on age, gender, and college year (Hussong et al. 2021), though more ethnically diverse by design (46% male; 22% African American, 5% Hispanic/Latino, 60% European American, 9% Asian, 6% multi-racial, and < 1% Native American/Alaskan Native or Pacific Islander or unknown).

In the overarching study, participants completed two lab-based visits (of 75–90 minutes each) separated by two weeks in which they gave consent, completed online surveys, and received a $20 and a $25 incentive, respectively. As they left the second visit, students were invited to participate in the Text Messaging Study if they had an Android phone or an iPhone with them. In a separate consent procedure, we informed participants that the study entailed presenting their own smart phone to the RA who would connect it to a secure, non-networked computer using a standard USB cord and download their past two weeks of text messages with all communicants with whom they had texted. Consistent with North Carolina law (N.C. Gen. Stat. Ann. § 15A-287; Rasmussen et al. 2012), the IRB waived consent for these communicants. Participants entered a drawing for four $100 cash prizes.

Students’ texts were downloaded behind a privacy screen using secure, for-pay software (MOBILedit Forensic Express) that allowed us to selectively download SMS text data. Downloads included phone numbers, timestamps, and text for all sent and received messages during the last two weeks. Participants provided phone numbers for their mothers, fathers, romantic partners, and up to three friends which were used to anonymize the text message data (phone numbers were replaced with a relationship identifier (i.e., mother, father) or a random identifier (i.e., person1, person2) for other phone numbers).

Due largely to changing technology during the study period across the many types of participants’ phones, we successfully downloaded text messages from only 267 of 528 consented participants (51% capture rate), yielding 569,172 texts over the 14 preceding days. The majority of unsuccessful captures were from Android phone users (due to operating system updates that resulted in incompatibility with our download software), which resulted in a preponderance of iPhone users in the final text message sample (Hussong et al. 2021). Selection analysis showed that, other than being more likely to have an iPhone, participants did not differ substantially from others in the overarching study on demographic and risk indicators (Hussong et al. 2021). In the current analysis, we included only those who exchanged at least one text message with at least one parent in the prior two weeks (89%; n = 238). We also eliminated texts exchanged in group messages, leaving a sample of 215 students who exchanged 21,381 text messages with mothers and 182 students who exchanged 6358 text messages with fathers. Downloads included text but no images (for privacy, though an 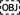 object replacement character flagged the presence of an indecipherable image; 3.8% of texts included an image). Simple emoticons (e.g., heart, smiley face) were captured intact but more complex emojis were downloaded as indecipherable symbols (4.4% of texts included an indecipherable emoji).

### 2.2. Measures

#### 2.2.1. Demographics

Emerging adults reported on their age, gender (male/female), and highest level of parent education (as a proxy for SES). They also self-reported on their race (1 item) and Hispanic/Latino ethnicity (1 item). For analyses here, we have re-coded these responses into three categories: Black (including one Afro-Latino emerging adult who endorsed Black race and Hispanic/Latino ethnicity), White (not Hispanic/Latino), and other race/ethnicity (which includes emerging adults who endorsed Hispanic/Latinx ethnicity alongside emerging adults who identified as American Indian or Alaskan Native, Asian, and Multiracial). Sample characteristics can be found in Table 1.

#### 2.2.2. Emerging Adult Perceived Parental Digital Pressure

In order to assess the ways in which parent-emerging adult text messaging might be associated with perceived parental intrusiveness (relevant to autonomy), emerging adults responded to ten items adapted from Hall and Baym’s (2012) measure of digital “entrapment” at the second lab visit. Items queried the extent to which emerging adults perceived intrusiveness, pressure and stress around parent-emerging adult contact by phone or online and perceptions that parents were annoyed when emerging adults were unavailable. We directed emerging adults to: “Please answer each of the questions below for your parent.” Thus, we cannot distinguish between perceptions of mothers and fathers. Response options ranged from 0 “Not at all true” to 4 “Extremely true.” An initial CFA showed that the ten items had a poor fit to a single factor model (χ^2^(35) = 122.105, *p* < 0.0001; TLI = 0.869, RMSEA = 0.104 [CI 0.084 to 0.124); SRMR = 0.063) with modification indices suggesting residual correlations among clusters of items (e.g., those that mentioned parental “annoyance”). Given the focus of the study, we dropped items focused on parent annoyance, emerging adult stress and emerging adult disengagement and retained four items tapping *parental digital pressure*; the resulting single factor model provided a good fit to the data (χ^2^(2) = 2.128, *p* = 0.345; TLI = 0.998, RMSEA = 0.017 [CI < 0.001 to 0.133); SRMR = 0.016). All items loading strongly on the digital pressure factor, including: “I feel pressured that I have to be available to this person by phone or online” (λ = 0.762; *M* = 1.087, *SD* = 1.244), “I feel pressured to text or post online to tell this person what I am doing” (λ = 0.761; *M* = 0.502, *SD* = 0.935), “I feel pressured to text or post online to keep in touch with this person” (λ = 0.862; *M* = 0.739, *SD* = 1.095), “I feel pressured to respond quickly to all texts or online posts from this person” (λ = 0.741; *M* = 0.765, *SD* = 1.124). Emerging adult perceived parental digital pressure was modeled as a latent variable in subsequent analyses.

#### 2.2.3. Emerging Adult Perceived Parental Text Supportiveness

To assess the ways in which parent-emerging adult text messaging is associated with perceived parent-emerging adult relatedness, emerging adults responded at the second visit to three items developed by the study team which queried how much they used text messaging to seek or receive parent support. Participants were told that: “The following are reasons why some people may use text messaging. Please indicate how true each reason is for you with regard to your text messaging using the following scale.” Response options ranged from 0 (“Not at all true”) to 4 (Extremely true”). The three items loaded strongly onto a single factor: “To get support from your parents for dealing with personal problems” (λ = 0.748; *M* = 1.001, *SD* = 1.125), “When you are feeling down or upset, to have your parents cheer you up” (λ = 0.940; *M* = 0.995, *SD* = 1.119), and “To get help, advice, or sympathy from your parents” (λ = 0.925; *M* = 1.040, *SD* = 1.126). The items asked about parents in general and did not distinguish between perceptions of mother and father separately. Emerging adult perceived parental supportiveness via texting was modeled as a latent variable in subsequent analyses.

#### 2.2.4. Parent-Emerging Adult Texting Frequency

The number of text messages exchanged between parents and emerging adults was computed directly from the captured text messages for each dyad (*M*_Mother Dyads_ = 102.84, *SD* = 139.52; *M*_Father Dyads_ = 36.69, *SD* = 49.95). Given the reciprocal nature of text messaging, the frequency of sent (from emerging adults) and received (to emerging adults) messages were highly correlated (*r*_Mother Dyads_ = 0.97, *r*_Father Dyads_ = 0.94) and thus we report the total frequency of mother-emerging adult and father-emerging adult text messages (a combined sum of sent and received). Emerging adults also self-reported their perceived parent-emerging adult texting frequency (i.e., “On a typical day, how much time do you spend interacting with your parents through texting - NOT including phone calls”) with response options of 0 = “I don’t use this” and 1 = “1 hour or less,” to 6 = “9 hours or more” (*M* = 1.248, *SD* = 0.650). This subjective perception of texting frequency with parents correlated *r* = 0.36, (*p* < 0.0001) with the total objective texting frequency count of sent and received text messages with mother and father combined.

#### 2.2.5. Parent-Child Text Interaction Coding Scheme (PCTICS)

Coding Manual Development. PCTICS codes were developed to tap theoretically relevant dimensions of parent-child interactions across monitoring and positive connection (see Table 1; Jensen 2017) and were adapted to fit the text-message medium from existing observational coding systems or survey measures. The coding manual included extensive examples and clarifications (of potentially tricky instances which would or would not meet the criteria for each code); key examples and clarifications are noted here. Micro-level coding occurred at the level of a single text and codes were not mutually exclusive or exhaustive (i.e., 42% of texts received no codes).

Coded domains tapping monitoring included emerging adult *disclosure*, parent *solicitation* of information, and parent exertion of *control* around rules and expectations for behavior; adapted from the dimensions identified by Kerr and Stattin (2000). Parent *solicitation* was coded whenever a parent’s text message queried the emerging adult’s behavior, wellbeing (e.g., health, sickness, mental health, sleep), activities, relationships, and whereabouts. Most solicitations took the form of questions, though some statements that were clearly intended to solicit information (e.g., “Grandma told me you were thinking about changing majors…”) were also assigned the solicitation code. Of note, solicitation was coded even if the query was about seemingly mundane topics; for example, a parent text of “What did you do today?” would be coded as a solicitation because it reflected an attempt to gain knowledge of the emerging adult’s activities. Not all parent questions were coded as solicitations; a guiding principle was that the question needed to serve to increase parent knowledge about the emerging adult’s wellbeing, behavior, relationships, or activities. Thus, questions about opinions, beliefs, and preferences (e.g., “What do you think of that new governor?”, “Do you like macaroni?”) would not be coded as solicitation. Examples of parent solicitation include: “What time did you get to bed last night?”, “What did you eat today?”, and “Who did you go to the party with last night?”.

Emerging adult *disclosure* was coded whenever an emerging adult’s text message disclosed information about their behavior, wellbeing, activities, relationships, whereabouts, or plans for the future. Disclosure texts could be either spontaneous or prompted by parent solicitation. Not all statements containing information were coded as emerging adult disclosures. For instance, if the emerging adult shared information for the purpose of coordination (e.g., “Meet me by the front door; I am downstairs”) it would not be coded as a disclosure. As with solicitation, statements about opinions, thoughts, and preferences would not meet the criteria for a code of disclosure. Disclosures *about other people* (as in gossip or chit chat, e.g., “I heard that Veronica got into Johns Hopkins”) would not be coded as disclosures. Examples of emerging adult disclosures include, “I made a B on my exam last week”, “I went shopping and bought some new shoes”, and “I have been staying up late studying and I fell asleep in class last night”.

Parent *control* was coded whenever a text message reminded the emerging adult of expectations or rules for behavior. Control was coded when a statement was unsolicited, directive, actionable (refers to a specific behavior), and/or conveyed a norm or expectation for behavior. These codes were distinct from those for advice provision, which was coded when advice was solicited, the parent guidance was non-directive (e.g., framed as suggestions or something to consider) or took a teaching tone. Examples of parent control include: “It’s important that you get at least eight hours of sleep”, “Make sure you call your grandmother today”, and “Why didn’t you text me back to let me know what time you would be home last night?”

Coded domains tapping positive connection included *warmth* (adapted from Melby and Conger 2001), *gratitude* (adapted from Froh et al. 2011) and different types of social support (emerging adult *seeking* and parent *provision* of *emotional/esteem support*, *instrumental support*, and *advice*; House et al. 1988; adapted from Hussong et al. 2001; Shadur et al. 2015). Codes of *warmth* were assigned in both parent and emerging adult text messages that included expressions of care, concern, support, or encouragement. Warmth was a fairly general code meant to capture most kind, responsive communication, and often included endearments, expressions of affection and love, warm greetings, and compliments. Examples of warmth include: “Praying for you today!”, “Love you!”, and “Can’t wait to see you this weekend!”

A code of *gratitude* was assigned whenever a text message (from parent or emerging adult) conveyed gratitude or thanks. Generalized gratitude that was not directed towards the other interactor was still coded as gratitude (e.g., “I am so grateful to have gotten that scholarship”). Examples of gratitude include “Thank you so much!!”, “You’re the best!” (in response to a gift or support), and “I don’t know what I would do without you”.

Emerging adult support seeking and parent support provision were coded separately for three domains of support (emotional/esteem, instrumental, and advice). Support seeking was coded when an emerging adult text message conveyed a desire or need for support (within each domain separately). Specifically, *emotional/esteem support seeking* was coded whenever a text message conveyed a desire for emotional or esteem support, which often included features of disclosing distressing emotions or requests for comfort. Emotional/esteem support seeking was coded both for direct requests for support (e.g., “I am feeling so sad today, can we talk on the phone later?”) and indirect support seeking (e.g., “I feel like such a failure”). Many of these texts also met criteria for an emerging adult disclosure code. Examples include: “I’m really worried about my final exams”, “Do you think I am smart enough to get that job?”, and “Carol just broke up with me.”

Emerging adult *instrumental support seeking* was coded for emerging adult text messages that sought tangible aid. Many of these were explicit requests (e.g., “Can you send me $100?”) but others were more subtle (e.g., “I am short on money for rent”). Requests for favors, money, or other tangible supports were coded as instrumental support seeking. Examples include, ““Will you look over my grad school applications?”, “I think I am out of meal swipes”, and “Could you take me to get my car serviced this weekend?”.

Emerging adult *advice seeking* was coded whenever a text message sought to elicit advice or guidance. This often took the form of a direct request for advice (e.g., “Do you think I should take summer classes?”) or a direct question (e.g., “How do I check my oil?”). Examples include: “Is studying abroad a good idea?”, “How many jobs should I apply to?”, and “Should I buy this dress?”

Parent support provision was coded when the parent offered or provided support (within each domain of emotional/esteem, instrumental, and advice separately). Specifically, *emotional/esteem support provision* was coded whenever a text message offered or provided emotional support (communicates caring, concern, sympathy, or understanding and attempts to comfort or console) or esteem support (communicates that the emerging adult is highly valued). Emotional/esteem support provision can be distinguished from warmth in that emotional/esteem support provision must have occurred in response to a stressor or need, whereas warmth need not. For example, the statement, “I love you” may just be coded as warmth (if said spontaneously) or both warmth and emotional/esteem support provision (if said in response to a disclosure of negative emotion by the emerging adult). Many instances of emotional/esteem support provision were also coded as warmth, but not all instances of warmth were coded as emotional/esteem support provision. Examples include: “Together we will make it through this”, “What a bummer!”, and “I understand how hard this is for you.”

Parent *instrumental support provision* was coded whenever a text message discussed the offer or provision of tangible aid and needed to occur in the context of a need or support seeking. Instrumental support provision included both offers (even if the coder couldn’t determine if the offer was followed through on) and provision of money, goods, and favors. Examples include: “I will send you $100 to get you through to your next paycheck,” “Dad has an old phone, we will send it to you since you broke yours,” and “I could talk to him for you.”

Parent *advice provision* was coded whenever a parent text provided guidance or advice to the emerging adult. As noted above, advice provision was sometimes difficult to distinguish from parent control, and thus specific guidelines were provided to coders to guide coding decisions. In some dyads, parent advice provision took the form of scaffolding or help in problem solving (e.g., Socratic questioning rather than outright advice**).** Texts which used scaffolding and questions to promote problem solving (e.g., evaluate pros and cons, consider all the relevant factors) were coded as advice provision. Examples of advice provision include: “If I were you, I might think about asking if rent will go up next year”, and “I think in North Carolina maybe you have to go to the Driver’s license office to get an in-state license before you can register your car.”

*Harsh/conflictual* messages were also coded (adapted from Melby and Conger 2001), defined as texts which conveyed hostile, angry, critical, disapproving, rejecting, or contemptuous behavior toward the emerging adult, but occurred infrequently (only 6 occurrences across nearly 30,000 text messages) and thus were dropped from analysis.

Text Message Preparation. Prior to coding, text messages were subjected to an identity-finder program which flagged instances of 9-digit (social security) and 16-digit (credit card) numbers, which were then cloaked in text content. Further, consistent with the IRB approval, the first author read through all of the nearly 30,000 text messages before coding and cloaked any other identifiable information embedded within the text content (e.g., participant first and last names, phone numbers). She also flagged/removed instances of group text messages., leaving just mother-emerging adult and father-emerging adult text threads for coding.

**Figure 1.**
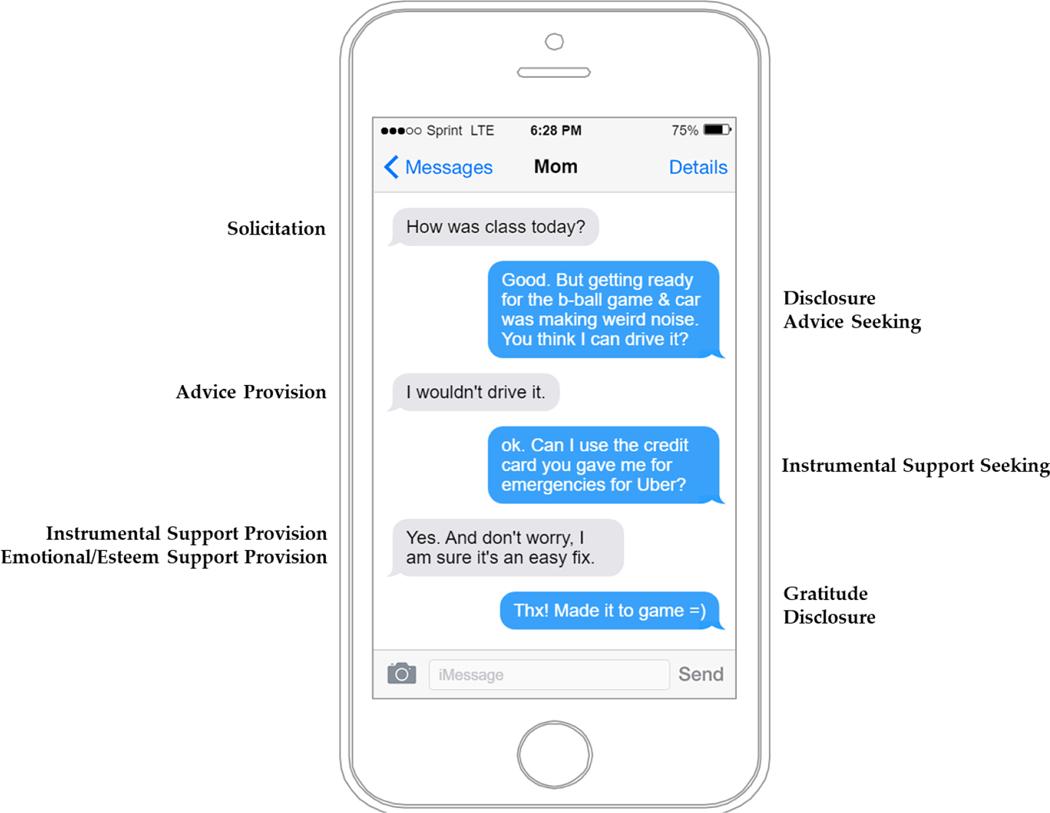
Simulated Text Message Conversation and PCTICS Codes.

Text Message Coding Procedures. Parent-emerging adult text messages were coded using Microsoft Access by an undergraduate coder who was trained to acceptable inter-rater reliability (IRR, Cohen’s kappa > 0.80) on an initial subset of the text message database with the first author (and code developer) for all PCTICS codes. Each text message was read in order and assigned as many codes (1 = present, 0 = absent) as applicable. As illustrated in Figure 1, codes were neither mutually exclusive nor exhaustive; many texts received multiple codes whereas others received none at all. For example, a parent text message stating “Good morning, honey! Did you get your paper turned in by the deadline last night?” would be assigned codes for both parental *warmth* and *solicitation*. Ultimately, 42% of text messages did not contain content that fell within the coded domains of monitoring or positive connection. Once baseline reliability was reached, previously coded text messages were re-coded by the newly reliable coder. To monitor coder drift and evaluate reliability, 20% of messages were double-coded. Final IRRs and percent agreement are reported in Table 2. According to Landis and Koch’s (1977) benchmarks for interrater reliability, most codes fell into the substantial (*Κ*s 0.61 to 0.80) to near perfect (*Κ*s 0.81 to 1.0) range, though one (parental instrumental support provision) evidenced only moderate (*Κ*s 0.41 to 0.60) agreement between raters. Overall, these interrater reliabilities are comparable to those seen in the Blackberry Project (Ehrenreich et al. 2020), with the lowest occurring for low-base rate behaviors (e.g., specific types of support provision).

### 2.3. Data Analyses

For Q1, the frequency of parent-emerging adult text interactions were computed separately for mother-emerging adult dyads (N = 215) and father-emerging adult dyads (N = 182) in SAS 9.4. Associations between mother- and father-emerging adult texting frequency and demographic covariates were tested in zero order correlations for continuous variables (age and parent-education) and in ANOVAs for categorical demographic covariates (gender and race/ethnicity).

For Q2, the frequency of each coded PCTICs domain was computed separately for mother-emerging adult texts interactions (N = 215) and father-emerging adult text interactions (N = 182) in SAS 9.4. Paired samples t-tests were used to compare the average frequency of each code for mother- and father-emerging adult dyads (among the sample of emerging adults who communicated with both a mother and a father; N = 159).

**Figure 2.**
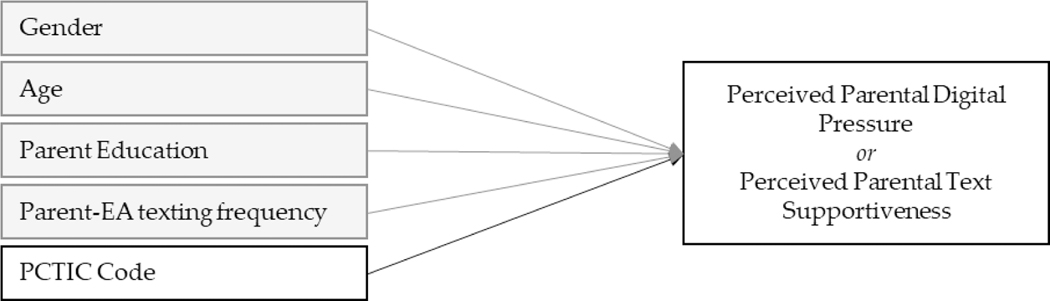
Q3 and Q4 Analytic Models. Note. Models were run separately for mother-emerging adult and father-emerging adult dyads. First, Perceived Parental Digital Pressure and Perceived Parental Text Supportiveness were regressed (in separate models) on parent-EA texting frequency and covariates of gender, age, and parent education (grey boxes/paths; results in upper panel of Tables 3 and 4). Next, Perceived Parental Digital Pressure and Perceived Parental Text Supportiveness were regressed (in separate models) on each PCTIC code separately, alongside covariates and parent-EA texting frequency (results in lower panel of Tables 3 and 4).

For Q3 and Q4, associations between frequency and content of text interactions and perceived digital pressure and perceived text supportiveness were tested using structural equation modeling performed with Mplus version 8 with MLR estimation (with robust standard errors) and full information maximum likelihood handling of missing data. Analyses were conducted separately for mother- and father-emerging adult dyads. First, as shown by the grey boxes/paths in Figure 2, emerging adult perceived parental digital pressure and perceived parental text supportiveness were regressed (in separate models) on parent-emerging adult text frequency, alongside covariates of gender, age, and parent education as a proxy for SES. Next, each PCTICS code was added to the above models (separately; black path) to allow for an assessment of the association of each individual code, over-and-above the associations with parent-emerging adult texting frequency and covariates.

## 3. Results

### 3.1. How Often Are Parent-Emerging Adult Dyads Text Messaging, and Are There Systematic Differences in Parent-Emerging Adult Texting Frequency? (Q1)

As seen in Table 3, emerging adults exchanged considerably more text messages with their mothers (*M* = 102.84 texts over the two-week study) than their fathers (*M* = 36.69; *t*(158) = −6.45, *p* < 0.001). Among those who texted with both a mother and a father (N = 159), the frequency of texting with mother and texting with father texting were only weakly correlated (*r* = 0.157, *p* = 0.048).

Overall, mother-emerging adult and father-emerging adult texting frequency were mostly similar across demographic groups. Mother-emerging adult texting frequency did not vary by the emerging adult’s age (*r* = −0.107, *p* = 0.149), *F*(2) = 1.93, *p* = 0.147), or gender (*F*(1) = 1.84, *p* = 0.176). Mother-emerging adult dyads from families characterized by a higher level of parent education (as a proxy for SES) tended to text message slightly more than those from families with a lower level of parental education (*r* = 0.134, *p* = 0.05). Father-emerging adult texting frequency also did not vary by the emerging adult’s age (*r* = −0.053, *p* = 0.437), race/ethnicity(*F*(2) = 1.94, *p* = 0.146), gender (*F*(1) = 0.05, *p* = 0.816), or parent education (*r* = 0.016, *p* = 0.534).

### 3.2. How Much Are Parent-Emerging Adult Dyads Using Text Messaging for Monitoring and Positive Connection, and Are There Differences in the Frequency of Text Messaging for These Purposes between Mother-Emerging Adult and Father-Emerging Adult Dyads? (Q2)

As seen in Table 3, mother-emerging adult texts were more frequent than father-emerging adult texts across all coded domains of monitoring and positive connection (*t*s (158) ranged 3.11 to 6.11, all *p*s < 0.002). Within both mother- and father-emerging adult dyads, the most common code was emerging adult disclosure (*M*_Mother Dyads_ = 19.33, *M*_Father Dyads_ = 6.19), followed by parent solicitations (*M*_Mother Dyads_ = 8.53, *M*_Father Dyads_= 2.94), and then parent warmth (*M*_Mother Dyads_ = 6.51, *M*_Father Dyads_ = 2.29). Several codes were quite infrequent, with a substantial proportion of dyads never evidencing the coded behaviors. For instance, 85% of emerging adults never texted a father and 67% never texting a mother seeking emotional/esteem support, and 84% of fathers and 60% of mothers never provided emotional/esteem support via text.

### 3.3. Are Parent-Emerging Adult Text Frequency and Content Related to Perceived Digital Pressure from Parents? (Q3)

Models testing associations between mother- and father-emerging adult texting frequency, PCTICS codes, and perceived parental digital pressure (alongside demographic covariates of age, gender, and parent education as a proxy for SES) all demonstrated good fit (Hu and Bentler 1999) to the data (χ^2^ *p* ranged 0.043 to 0.74; RMSEA ranged < 0.001 to 0.60; SRMR ranged 0.018 to 0.034).

Emerging adults who exchanged more texts with their mothers reported stronger perceived parental digital pressure (after controlling for emerging adult gender, age, and parent education; Table 4). Once associations with mother-emerging adult texting frequency were taken into account, emerging adult perceptions of parental digital pressure were unrelated to most forms of positive connection and monitoring, with two exceptions. Those emerging adults who engaged in more disclosures and who displayed more gratitude in their text messages to mothers reported perceiving significantly less parental digital pressure.

In contrast to mother-emerging adult dyads, the frequency of father-emerging adult text interactions was unrelated to emerging adult perceptions of parental digital pressure (Table 4). All father-emerging adult codes were unrelated to emerging adult perceptions of digital pressure.

### 3.4. Are Parent-Emerging Adult Text Frequency and Content Related to Perceived Text Message Support from Parents? (Q4)

Models testing associations between mother- and father-emerging adult texting frequency, PCTICS codes, and perceived parental text message support (alongside demographic covariates of age, gender, and parent education as a proxy for SES) all demonstrated good fit (Hu and Bentler 1999) to the data (χ^2^ *p* ranged 0.100 to 0.968; RMSEA ranged < 0.001 to 0.057; SRMR ranged 0.014 to 0.022).

As seen in Table 5, emerging adults who exchanged more text messages with their mothers reported higher levels of perceived digital supportiveness from parents. Once this effect of mother-emerging adult texting frequency was taken into account, only instrumental support seeking was significantly associated with emerging adult perceptions of parental text message supportiveness. Those emerging adults who engaged in *more* instrumental support seeking via text message tended to report that they used text messaging *less* for seeking out and receiving emotional support from their parents.

Among fathers, in contrast, overall texting frequency was not significantly associated with emerging adult perceived parental text supportiveness; that is, emerging adults who texted more with their fathers were no more or less likely to report that they used text messages to seek emotional support from parents. Instead, several significant associations between perceived parental text support and content codes of father-emerging adult interactions emerged. As seen in Table 4, those emerging adults who expressed more frequent gratitude in their text messages to fathers tended to report that they used text messaging more often for support seeking and receipt. As hypothesized, those emerging adults who engaged in more emotional/esteem support seeking and whose fathers provided more emotional/esteem support via text tended to self-report being heavier users of text messaging for emotional support seeking/receipt. A similar association emerged for emerging adult advice seeking and father advice provision, such that more advice seeking and provision were associated with higher perceived parental text supportiveness.

## 4. Discussion

Emerging adult college students and their parents vary widely in how often they exchange text messages and in the way they use text messaging to build positive connections and to enact monitoring and disclosing behaviors. Anticipated differences in texting patterns were evident in exchanges with mothers versus fathers, though college students were generally not very reliable in reporting the frequency of their own texting behaviors. Importantly, common parenting behaviors involving positive connection and monitoring and disclosing behavior were evident in the text message exchanges of parents and emerging adults. Moreover, evidence of parents’ and emerging adults’ contributions to these “parenting” behaviors supported a more dyadic view of parenting in this developmental period. Finally, we found some evidence that these dyadic parenting behaviors were associated with digital analogues to autonomy and relatedness to parents in emerging adults. These findings are unique given the methodology of the current study. The examination of the frequency and content of parent-emerging adult digital interactions gives us a direct window into real-time parent-emerging adult communication that is longer and less contrived than traditional observational paradigms and less subject to the biases of self-report surveys. Our analyses of the nearly 30,000 naturally occurring text-message interactions between college students and their parents over a two-week period offered several important observations which we consider in turn.

### 4.1. The Nature of Text Message Exchanges between Parents and Emerging Adults

College students and their parents are in frequent text message contact, with considerable variability in the extent and nature of this contact. Direct examination of text message threads shows more frequent contact with mothers (an average of about 8 texts per day) than with fathers (an average of about 3 texts per day). Somewhat surprisingly, given concern about a digital divide in unequal access to and use of smartphone and digital technologies (Norris 2001; George et al. 2020) and past evidence of more frequent parent-adolescent digital communication among older and female adolescents (Jensen et al. 2021; Rudi et al. 2015), the frequency of parent-emerging adult text message communication did not differ by emerging adult gender, age, or race/ethnicity. Indeed, the only significant demographic difference that emerged was that emerging adults whose parents had higher levels of educational attainment exchange slightly more messages with mothers (but not fathers) compared to their peers. This suggests that there may be more similarities than differences across demographic groups in the frequency of objectively assessed parent-emerging adult texting frequency, though emerging adults and mothers with higher socioeconomic status may be slightly more likely to text message. It is also worth mentioning that this sample (of college students from an elite public university) had parents who were on average highly educated (almost half had a parent with a graduate degree, and nearly 80% had graduated college) and thus findings may not be representative of or generalize to the entire range of educational backgrounds and socioeconomic statuses present in other college settings or among non-college attending emerging adults. Within this caveat, however, these findings suggest that digital parenting shares similar characteristics across college students from varying backgrounds.

Consistent with co-construction theory’s assertion that emerging adults’ online worlds are “psychologically continuous” with their offline world (Subrahmanyam et al. 2008, p. 421), our findings on parent gender differences in mother- and father-emerging adult digital interactions closely correspond to gender differences observed in traditional face-to-face parent-youth interactions (Nelson et al. 2011; Smetana et al. 2006; Keijsers and Poulin 2013; Son and Padilla-Walker 2021). That is here, mother-emerging adult dyads were significantly more likely than father-emerging adult dyads to display positive connection and monitoring and disclosing behaviors in their text message exchanges. This is also consistent with recent research where youth self-report more frequent mother than father digital interactions (Miller-Ott et al. 2014; Fingerman et al. 2012). In addition, our finding of parent gender differences in objectively measured parent-emerging adult texting frequency complements past self-report survey research in which emerging adults reported that there were stronger rules, norms and expectations around when and for what purposes they could or should text their fathers than mothers (Miller-Ott et al. 2014), which the authors interpreted as evidence of greater mother availability by phone. These findings also parallel research with younger children that suggest that the majority of child caretaking activities continue to fall to mothers over fathers (Craig 2006) and that this pattern of gendered parenting continues even into emerging adulthood.

The most common “parenting” behavior in these text exchanges was disclosures by emerging adults, followed by parental solicitations. This finding may suggest that following Kerr and Stattin (2000), the knowledge that parents use to monitor child behavior is more often gathered by child disclosures rather than by parent solicitation. This pattern likely extends from those established in adolescents and emphasizes the importance of fostering communication patterns, on or offline, that create a safe and supportive space in which youth feel comfortable disclosing to parents. That such patterns continue into emerging adulthood in a digital context is perhaps not surprising given that adolescent reports that texting is a convenient way to update parents on their location and activities (Fletcher et al. 2018).

Texts around positive connection (especially expressions of support seeking and provision) occurred relatively infrequently. Despite their infrequence within text message streams, the exchange of texts about positive connection were still evident within most dyads; most mothers and fathers expressed warmth toward their emerging adults over the study period and about half of youth did so toward parents. Notably, 52% of dads and 77% of mothers provided support via text (whether emotional esteem, instrumental, or advice) over the two-week study period and 66% of youth sought support from mothers and 44% from fathers. This is consistent with our recent self-report research among younger adolescents (Jensen et al. 2021), where text-based social support was relatively uncommon but quite variable across dyads. Moreover, this finding once again reflects the dyadic nature of support in parent-emerging adult relationships. To better understand how support is most effectively delivered (e.g., whether in response to youth bids for support, whether as matched in type to that requested by youth), further analyses are needed.

### 4.2. Associations between Digital Parenting and Analogue Milestones in Emerging Adulthood

Although inconsistent, we found evidence that the content of text exchanges between parents and emerging adults is related to digital analogues of the developmental milestones of autonomy and relatedness. More specifically, the more frequently emerging adults texted with their mothers, the more likely they were to report feeling pressure to be available and responsive to parents online. The same association, however, was not apparent in emerging adults’ interactions with fathers. Given that fathers and emerging adults exchanged far fewer text messages than mothers and emerging adults, father-emerging adult texting may not be as strongly tied to perceived parental digital pressure, especially given that our measure of digital pressure (which was worded about “your parent”) did not distinguish between perceptions of mothers and fathers. In fact, higher mother-emerging adult texting frequency may have made this dyad more salient when emerging adults were self-reporting on perceived digital pressure. This parent gender difference may also be consistent with past findings that father-emerging adult digital communications are characterized by stronger boundaries and rules (e.g., around time and extent of availability) than mother-emerging adult digital communications (Miller-Ott et al. 2014) and thus perhaps present fewer opportunities for intrusion.

In previous studies digital pressure from peers has been associated with less friendship satisfaction (Hall and Baym 2012). Our findings indicate the digital pressure may also be relevant in the parent-youth relationship, though overall low levels of perceived parental digital pressure suggest that many youths are not overly concerned or bothered by their parents’ digital communications (similar to past research suggesting high levels of emerging adult satisfaction with parent cell phone contacts; Miller-Ott et al. 2014).

Although the frequency of mother-emerging adult interactions was related to more perceived digital pressure, most coded domains of parent-emerging adult positive connection and monitoring (even those we hypothesized would be the most strongly linked with perceptions of parents as intrusive: parental solicitation and control) were not related to emerging adult perceptions of digital pressure. Indeed, the only texting behaviors that were associated with perceived digital pressure were the extent to which emerging adults made disclosures and conveyed gratitude in their text messages with mothers (both of which were linked to lower perceived parental digital pressure). These results suggest that emerging adult perceptions of intrusiveness and pressure to engage digitally are perhaps primarily driven by the frequency (rather than the content) of parent text messaging, though perhaps (not surprisingly, and consistent with past self-report studies on youth-driven cell phone communications; Weisskirch 2009, 2011) certain types of emerging adult-driven communications may be valued and perceived positively (and not as intrusive) by emerging adults.

These results may inform theories about the nature of positive connection and monitoring and disclosing behaviors during emerging adulthood. In particular these results draw into question the accuracy of modern theories on “helicopter parenting” which often assert that Millennial and Gen Z youth are over-monitored and over-supported/coddled (with frequent digital communication cited as one tool for overparenting; Padilla-Walker and Nelson 2012; Jiao and Segrin 2021). It may be that emerging adults and their parents have established new norms for parent-emerging adult engagement in which even monitoring (traditionally seen as developmentally inappropriate at this stage) does not have to be intrusive or autonomy inhibiting when conceptualized as a bi-directional, transactional process in which parents ask developmentally appropriate questions to check in about the child’s wellbeing, and the child chooses to disclose in turn.

Interestingly, not only did youth who texted more often with mothers tend to report greater parental digital pressure, they also tended to endorse more experiences of parental text-based supportiveness. This is in line with recent findings that those adolescents who self-report texting more with parents also report having a closer parent-adolescent relationship (Manago et al. 2020). Emerging adults who engaged in more instrumental support seeking also tended to endorse *less* perceived parental text support. This is opposite of the hypothesized association but may highlight the importance of distinguishing between different types of support parents enact (i.e., emotional/esteem vs. instrumental vs. advice) and the extent of support emerging adults perceive in their relationships with parents. An important future direction for this research will be to examine match/mismatch (Wang 2019) of emerging adult expectations (i.e., quantity and type of support seeking) and parent provision (both quantity and type) of support; it may be that it is not the absolute quantity of support provision that matters so much as the extent to which it is appropriately responsive to emerging adult expectations and needs. Moreover, given the cross-sectional design, it is unclear if parents offering support decreases bids for support in youth as well as distress versus parents offer more support in response to youth distress and bids for support. Prospective studies are needed to untangle these mechanisms.

In contrast, father-emerging adult texting frequency was not associated with perceived parental text message support. It is again possible that this lack of association in father-emerging adult dyads may be driven by greater texting frequency with mothers making that relationship more salient when emerging adults responded to the parental text supportiveness measure which asked about “your parents” rather than mothers and fathers separately. However, several codes emerged as important correlates of perceived parental text support. Emerging adults whose texts to fathers included more gratitude, emotional/esteem support seeking, and advice seeking reported having parents who were more supportive via text message. Similarly, fathers who provided more emotional/esteem support and advice had emerging adults who reported more perceived parental support. This underscores the validity of objectively coded parent-youth text interactions, and highlights that, even though they are infrequent, supportive father-emerging adult text message interactions may reflect a stronger parent-emerging adult relationship. These differences in mother and father dyads may also suggest that in mother-emerging adult dyads (where contact is more frequent) the frequency of contact may more strongly impact the nature of the underlying relationship, whereas in father-emerging adult dyads (where text contact is much less frequent) the content shines through. As much past research has not parsed the differences between mother and father dyadic text communication (Manago et al. 2020; Ehrenreich et al. 2020), it will be important for future research to consider parental gender difference in the frequency and potential impact of parent-emerging adult text messaging.

### 4.3. Methodological Observations

As suggested in the literature (Gold et al. 2015; Parry et al. 2021), emerging adults in the current study were not very accurate at estimating how much they text with parents, with the number of texts exchanged with parents being only modestly correlated (*r* = 0.36) with self-reported frequency of parent text message contact by emerging adults. This highlights the importance of moving beyond self-reported frequency of digital contact and towards objective assessment using tools like billing records and device logs. Here, the examination of quantitative meta data (on objective parent-emerging adult text frequency) and qualitative codes of parent-emerging adult interactions tapping positive connection and monitoring suggest that text messages are a rick source of information about the content of parent-emerging adult digital exchanges.

Results also underscore, however, some of the challenges and barriers to operationalizing nuanced dimensions of the parent-emerging adult relationship within the content of text messages. There was substantial variability in interrater agreement amongst codes. In particular, interrater reliability for some low base rate codes indexing emerging adult seeking and parental provision of different types of support fell below Kripendorff’s (1980, 2018) recommended cutoff of 0.67 for drawing valid conclusions from content analysis. Importantly, high rates of inter-rater agreement which are less impacted by low base rates (all above 87%) were reported. Our experience here is that it is in fact quite difficult to train coders to perfect agreement on the nuanced distinction between different types of social support, especially as they manifest within brief (sometimes single word or short phrase) text messages and without the benefit of context clues and nonverbal signals. For example, coders sometimes struggled to parse distinctions like whether a text in which a mother gave detailed feedback on a college essay was more consistent with instrumental support (doing the child a favor by reading their essay) and/or informational support/advice. Nonetheless, we think even these lower reliability codes have value, given that they are quite consistent with the magnitude of interrater agreement in the only other study of parent-child text message content (Ehrenreich et al. 2020). In addition, these metrics of reliability focus on interrater agreement at the level of individual text messages, but when we consider interrater agreement at the level of the parent-emerging adult dyad over the course of the two-week study period (the level of analysis in the current study) interrater agreement rates (correlations) are exceedingly high (> 0.98). Future analyses that focus on smaller time scales within text-message analyses (within days or day-to-day exchanges), may best consider superordinate domains of interest (i.e., positive connection and monitoring and disclosing behaviors) for which more acceptable reliability estimates were evident (*Κ*_EA positive connection_ = 0.78, *Κ*_Parent positive connection_ = 0.74, *Κ*_Parent monitoring_ = 0.78). It must also be noted that low interrater reliability can increase the likelihood of Type II errors (false negatives), and thus it is possible that improved measurement of these constructs might reveal additional associations.

### 4.4. Strengths, Limitations, and Future Directions

Strengths of this study include direct observation of digital communication rather than self-reports of such behaviors, inclusion of fathers and mothers, and consideration of offline and online dimensions of these parent-emerging adult relationships. Still, this study has several limitations that deserve consideration. First, despite the rich, intensive-longitudinal nature of the observational text message data, the analyses conducted here are essentially cross-sectional. Future longitudinal research is needed to disentangle the temporal associations between parent-emerging adult digital engagement and features of the parent-emerging adult relationship. Second, this study captured a short snapshot (2 weeks) of only one platform for parent-emerging adult interaction and did not allow for analysis of interactions on other technologically mediated platforms (e.g., phone calls, social media, other private messaging applications) nor interactions occurring face-to-face. With the rapidly changing face of digital technology, this is likely to always be a challenge, though the fundamental methods and core constructs presented here provide a basis upon which to advance this evolving literature. Third, reports of emerging adult perceived parental digital pressure and parental text support did not distinguish between separate perceptions of mothers and fathers. Despite these limitations, this study lays the groundwork for future research which can more finely parse perceptions of mothers and fathers (and ideally, other parent figures and caregivers) alongside the content of dyadic digital communications.

## 5. Conclusions

### 5.1. Implications for 21st Century Parenting and Parent-Emerging Adult Relationships

The present study has implications for parenting, education and practice with emerging adults in the 21st century. Parents remain a salient part of emerging adults’ social networks during the college years and digital communication is a core platform for parent-youth interactions in the modern era. Results here suggest that text messaging serves diverse purposes, including positive connection as well as monitoring and disclosing. Moreover, traditional developmental tasks, such as maintaining relatedness while establishing autonomy, may play out online as well as offline. For college students not living at home, digital communication may thus serve as an important tool for navigating these tasks in emerging adulthood. Educators and practitioners working with emerging adults (e.g., college administrators, mental health and career counselors) would do well to consider the ongoing and important role of parents in emerging adults’ healthy psychosocial development and to leverage parent (digital and face to face) supports as potential assets.

### 5.2. Implications for Future Research

Results here can also greatly inform future research. Given the low correlation between perceptions of text messaging frequency and actual recorded texting behaviors, these analyses demonstrate the potential advantages of directly observing naturally occurring parent-emerging adult interactions through an increasingly used communication platform, particularly for those living away from home. In addition to qualitative coding schemes (such as the PCTICS) to capture the content of parent-youth text messages, researchers can learn much about relationship dynamics from indices derived from meta-data (e.g., frequency, timing, latency to response), quantitative analysis of words using established and newly developed dictionaries (Jensen and Hussong 2021), and machine learning approaches. In short, the potential for analysis of text message data to explore relationship dynamics is remains to be fully tapped. Although many of these applications have been applied to public-facing social networking site content, further consideration of what happens in parent-emerging adult private messaging is likely to provide novel insights. This burgeoning field is just beginning. The current study adds to the needed theoretical and methodological work for not only understanding relationships between parents and emerging adults in the digital era but also for leveraging digital communication as a platform for prevention and intervention.

## Figures and Tables

**Table 1.** Sample Characteristics of Parent-Emerging Adult Dyad Text Message Sample (*N* = 238).

Demographics			% of Sample	Mean (SD)
Age				19.85 (1.39)
Male Gender			39.08%	
Race				
	Black/African American		21.01%	
	White (not Hispanic/Latino)		56.72%	
	Other Race/Ethnicity		22.27%	
		Latino/Hispanic	7.14%	
		American Indian, Alaska Native	0.84%	
		Asian	5.46%	
		Pacific Islander	0%	
		Multiracial	2.94%	
Parental Education				4.73 (1.36)
	Less than High School (1)		1.26%	
	High School Graduate (2)		4.2%	
	Some College or Technical School (3)		14.71%	
	College Graduate (4)		28.15%	
	Some Graduate, Medical, or Professional School (5)		3.78%	
	Completed Graduate, Medical, or Professional School (6)		47.9%	

**Table 2.** Interrater Reliability.

Code		IRR (Κ)	% Agreement
*Monitoring*			
	EA Disclosure	0.73	0.87
	Parent Solicitation	0.82	0.96
	Parent Control	0.69	0.97

*Positive Connection*			
	EA Warmth	0.82	0.98
	Parent Warmth	0.83	0.96
	EA Gratitude	0.96	1.0
	Parent Gratitude	0.96	1.0
	EA Emotional/Esteem Support Seeking	0.73	0.98
	Parent Emotional/Esteem Support Provision	0.66	0.98
	EA Instrumental Support Seeking	0.63	0.98
	Parent Instrumental Support Provision	0.54	0.96
	EA Advice Seeking	0.75	0.99
	Parent Advice Provision	0.61	0.96

Note. N = 238 emerging adults. EA = Emerging Adult.

**Table 3.** Descriptive Statistics for coded PCTICS domains.

		Mother-Emerging Adult Dyads (*N* = 215)				Father-Emerging Adult Dyads (*N* = 182)			

		*M*	*SD*	% w/ 0	Max	*M*	*SD*	% w/ 0	Max
Parent-EA Texting Frequency		102.84	139.52	--	1012	36.69	49.95	--	501

Monitoring									
	EA Disclosure	19.33	27.84	13	177	6.19	13.19	21	163
	Parent Solicitation	8.53	13.03	16	118	2.94	8.74	38	113
	Parent Control	2.98	6.20	45	50	1.02	2.32	65	15

Positive Connection									
	EA Warmth	3.13	5.43	39	47	1.21	3.17	60	32
	Parent Warmth	6.51	10.49	21	83	2.29	4.18	41	41
	EA Gratitude	2.12	3.87	38	35	0.93	1.59	55	13
	Parent Gratitude	0.92	1.57	56	13	0.45	1.02	74	7
	EA Emotional/Esteem Support Seeking	1.88	5.84	67	47	0.30	.92	85	7
	Parent Emotional/Esteem Support Provision	2.09	5.93	60	58	0.36	1.20	84	10
	EA Instrumental Support Seeking	1.60	2.97	50	26	0.65	1.40	71	8
	Parent Instrumental Support Provision	2.59	4.17	37	31	0.93	2.00	63	15
	EA Advice Seeking	1.22	3.07	67	24	0.44	1.37	79	12
	Parent Advice Provision	2.50	5.88	54	49	0.83	2.34	73	20

Note. EA = Emerging Adult. Means (*M*) and standard deviations (*SD*) reported acros all dyads over the entire 2-week study period alongside the percent of the sample who evidenced no instances of the code (% w/ 0) and the maximum frequency of each code (Max) to capture the range.

**Table 4.** Associations Between Parent-Emerging Adult Texting Frequency and Content Codes with Emerging Adult Perceived Parental Digital Pressure.

		Perceived Parental Digital Pressure							

		Mother-Emerging Adult Dyads (*N* = 215)				Father- Emerging Adult Dyads (*N* = 182)			

		*b*	*SE*	*p*	*β*	*b*	*SE*	*p*	*β*
Gender (male)		0.069	0.152	0.644	0.034	−0.013	0.164	0.936	−0.006
Age		−0.074	0.047	0.118	−0.104	−0.057	0.053	0.281	−0.080
Parent Education		−0.073	0.058	0.207	−0.103	−0.083	0.073	0.258	−0.106
Parent- EA texting frequency		**0.001**	**0.001**	**0.035**	**0.194**	0.002	0.002	0.351	0.075

Monitoring									
	EA Disclosure	**−0.017**	**0.006**	**0.008**	**−0.474**	−0.018	0.015	0.232	−0.231
	Parent Solicitation	−0.009	0.011	0.382	−0.126	−0.004	0.017	0.795	−0.038
	Parent Control	−0.009	0.020	0.650	−0.058	0.102	0.056	0.070	0.238
Positive Connection									
	EA Warmth	0.001	0.025	0.970	0.005	0.028	0.034	0.399	0.090
	Parent Warmth	<0.001	0.013	0.988	−0.002	0.029	0.025	0.239	0.123
	EA Gratitude	**−0.045**	**0.019**	**0.015**	**−0.180**	0.047	0.049	0.332	0.075
	Parent Gratitude	−0.008	0.053	0.875	−0.014	−0.009	0.091	0.921	−0.009
	EA Emotional/Esteem Support Seeking	−0.023	0.015	0.111	−0.140	0.024	0.105	0.818	0.022
	Parent Emotional/Esteem Support Provision	−0.020	0.022	0.373	−0.121	−0.005	0.070	0.947	−0.006
	EA Instrumental Support Seeking	−0.033	0.029	0.263	−0.100	0.012	0.076	0.873	0.017
	Parent Instrumental Support Provision	−0.026	0.023	0.270	−0.111	−0.038	0.066	0.565	−0.076
	EA Advice Seeking	0.017	0.038	0.649	0.055	−0.057	0.061	0.349	−0.079
	Parent Advice Provision	−0.002	0.018	0.932	−0.009	−0.033	0.054	0.547	−0.076

Note. The upper panel includes presents results of the initial structural equation model, which tested associations between parent-EA texting frequency with Perceived Parental Digital Pressure, alongside covariates of gender, age, and parent education (separately for Mother-Emerging Adult and Father-Emerging Adult dyads). The lower panel includes results from subsequent models, which added each PCTIC code to the model (which already included covariates and parent-EA texting frequency) separately. EA= Emerging Adult. Raw regression coefficients (*b*), standard errors (*SE*), *p* values (bolded when *p* < 0.05), and standardized regression coefficients (*β*) presented.

**Table 5.** Associations between Parent-Emerging Adult Texting Frequency and Content Codes with Emerging Adult Perceived Parental Text Support.

		Perceived Parental Text Supportiveness							

		Mothers (N = 215)				Fathers (N = 182)			
		
		*b*	*SE*	*p*	*β*	*b*	*SE*	*p*	*β*
		
Gender (male)		**−0.472**	**0.127**	**<0.001**	**−0.268**	**−0.487**	**0.146**	**0.001**	**−0.280**
Age		−0.052	0.039	0.182	−0.084	**−0.091**	**0.043**	**0.035**	**−0.148**
Parent Education		−0.012	0.048	0.799	−0.020	0.044	0.061	0.468	0.066
Parent-EA texting frequency		**0.001**	**<0.001**	**0.016**	**0.180**	0.001	0.001	0.488	0.050

Monitoring									
	EA Disclosure	0.001	0.006	0.817	0.042	−0.008	0.011	0.474	−0.126
	Parent Solicitation	0.001	0.008	0.902	0.015	−0.017	0.012	0.119	−0.189
	Parent Control	0.012	0.014	0.375	0.090	−0.031	0.029	0.278	−0.084
Positive Connection									
	EA Warmth	0.017	0.018	0.362	0.106	0.025	0.021	0.229	0.094
	Parent Warmth	−0.010	0.009	0.288	−0.122	0.012	0.015	0.453	0.056
	EA Gratitude	0.004	0.017	0.813	0.018	**0.105**	**0.037**	**0.005**	**0.196**
	Parent Gratitude	−0.002	0.034	0.943	−0.004	−0.077	0.051	0.130	−0.093
	EA Emotional/Esteem Support Seeking	−0.013	0.012	0.273	−0.087	**0.142**	**0.066**	**0.030**	**0.152**
	Parent Emotional/Esteem Support Provision	−0.010	0.011	0.357	−0.070	**0.144**	**0.036**	**<0.001**	**0.203**
	EA Instrumental Support Seeking	**−0.044**	**0.021**	**0.040**	**−0.153**	−0.015	0.054	0.780	−0.025
	Parent Instrumental Support Provision	−0.031	0.019	0.115	−0.151	−0.008	0.035	0.831	−0.017
	EA Advice Seeking	−0.031	0.021	0.139	−0.111	**0.095**	**0.039**	**0.014**	**0.153**
	Parent Advice Provision	−0.015	0.012	0.199	−0.104	**0.070**	**0.032**	**0.027**	**0.192**

Note. The upper panel includes presents results of the initial structural equation model, which tested associations between parent-EA texting frequency with Perceived Parental Text Supportiveness, alongside covariates of gender, age, and parent education (separately for Mother-Emerging Adult and Father-Emerging Adult dyads). The lower panel includes results from subsequent models, which added each PCTIC code to the model (which already included covariates and parent-EA texting frequency) separately. EA = Emerging Adult. Raw regression coefficients (*b*), standard errors (*SE*), and standardized regression coefficients (*β*) presented alongside *p* values (bolded when *p* < 0.05).
